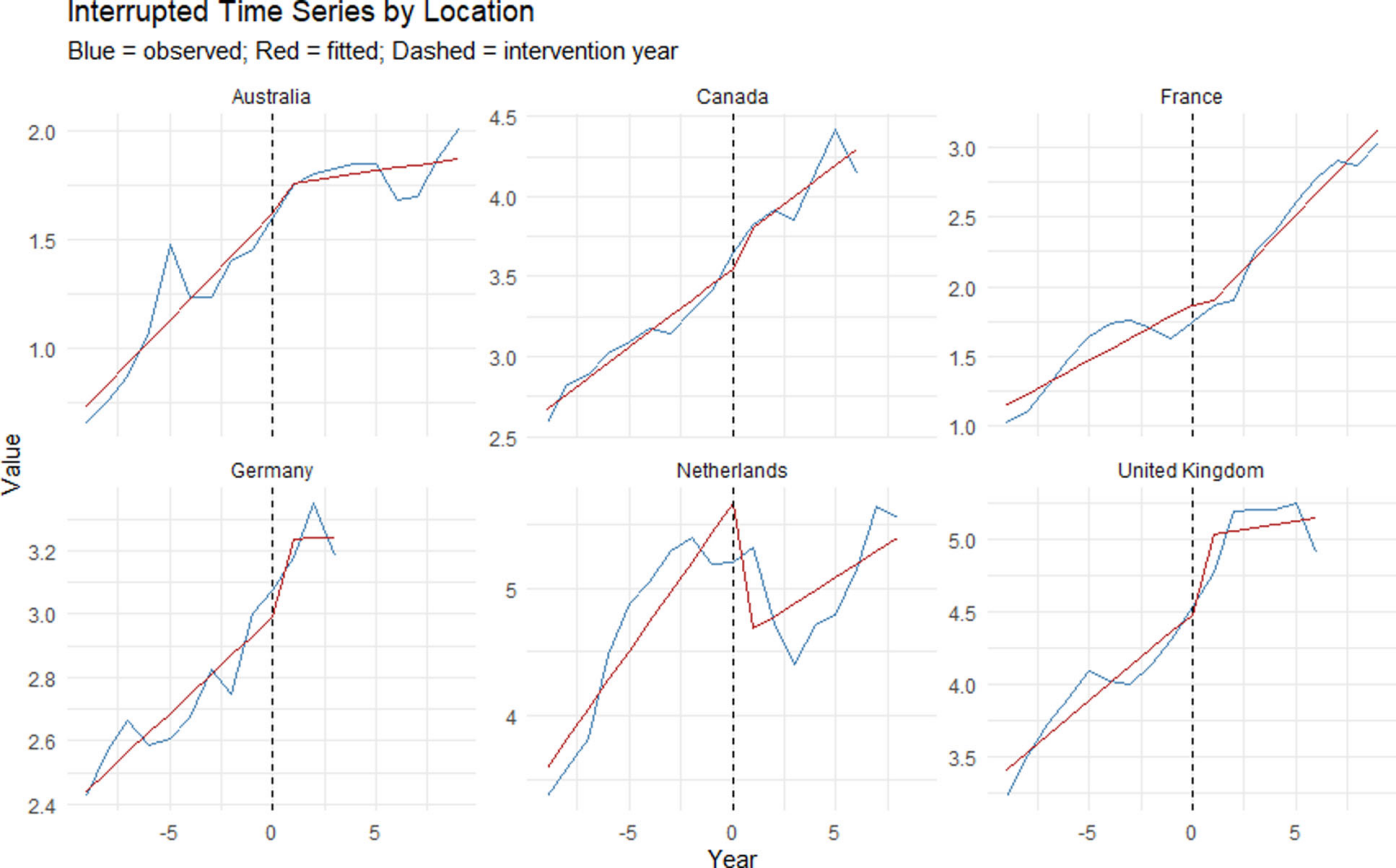# 43 Effect of CLABSI care bundle on reducing catheter line associated bloodstream infection in Cho Ray hospital in Vietnam

**DOI:** 10.1017/ash.2026.10481

**Published:** 2026-06-23

**Authors:** Kevin Ikuta

**Affiliations:** ^1^ Greater Los Angeles VA

## Abstract

**Background:** Legionella is the etiologic agent of Legionnaire’s disease and Pontiac fever with an environmental niche in potable water systems. Globally, Legionella mortality is estimated to have increased fourfold since 1990. Several regulations have emerged to try and counteract this increase, with six countries (Netherlands, Germany, England, France, Australia, and Canada) enacting laws that target the growth of legionella from environmental samples for primary prevention. The effectiveness of these laws in mitigating legionella mortality has not been evaluated. We performed an interrupted time series analysis to evaluate the effect of laws with specific colony forming unit (CFU) thresholds on the legionella mortality rate in the country. **Methods:** We extracted legionella mortality rate estimates produced by the Global Burden of Disease project for the six countries with legionella laws with defined CFU thresholds for the ten years before the laws were enacted and ten years after the laws were enacted (or up to 2020). We then performed an interrupted time series analysis for these six countries. All analysis was performed in R version 4.5.2. **Results:** The median legionella mortality rate for the six countries included in analysis in the decade before laws were enacted was 2.86 deaths per 100,000 (IQR1.6-3.75). In the decade following law enactment the mortality rate was 3.35 legionella deaths per 100,000 (IQR 2.01-4.77). In the interrupted time series, there was an increase in legionella mortality of 0.17 deaths per 100,000 (95%CI 0.14 – 0.2) per year in the decade before CFU laws were introduced. The rate of increase did not statistically significantly change after law enactment with an annual increase of 0.102 per 100,000 (95%CI 0.03 – 0.17) persons. A country specific analysis found that the rate of annual increase in legionella mortality rates was statistically significantly lower following enactment of CFU specific laws in Germany, Netherlands, and the United Kingdom (Figure1). Discussion: We found that, collectively, laws including CFU thresholds had no significant effect on the rate of change in legionella mortality. However, at the individual country level, three countries did see a significant decrease in the rate of change in legionella mortality following enactment. One possible explanation for this heterogeneity is that several of these laws include additional mitigation measures and may confound the effect of the CFU based monitoring. Future studies should focus on these additional mitigation measures and compare legionella outcomes in countries with and without CFU thresholds in water safety laws.

**Figure:**